# How to develop a program to increase influenza vaccine uptake among workers in health care settings?

**DOI:** 10.1186/1748-5908-6-47

**Published:** 2011-05-19

**Authors:** Ingrid Looijmans-van den Akker, Marlies E Hulscher, Theo JM Verheij, Josien Riphagen-Dalhuisen, Johan JM van Delden, Eelko Hak

**Affiliations:** 1 Julius Center for Health Sciences and Primary Health Care, University Medical Center Utrecht, HP 6.131, POBOX 85500, 3508 GA Utrecht, The Netherlands; 2 Scientific Institute for Quality of Healthcare (IQ Healthcare), Radboud University Nijmegen Medical Centre, POBOX 9101, 114 IQ Healthcare, 6500 HB Nijmegen, The Netherlands; 3 University of Groningen, Department of Pharmacy, Pharmacoepidemiology and Pharmacoeconomy, A. Deusinglaan 1, 9713 AV, Groningen, The Netherlands

## Abstract

**Background:**

Apart from direct protection and reduced productivity loss during epidemics, the main reason to immunize healthcare workers (HCWs) against influenza is to provide indirect protection of frail patients through reduced transmission in healthcare settings. Because the vaccine uptake among HCWs remains far below the health objectives, systematic programs are needed to take full advantage of such vaccination. In an earlier report, we showed a mean 9% increase of vaccine uptake among HCWs in nursing homes that implemented a systematic program compared with control homes, with higher rates in those homes that implemented more program elements. Here, we report in detail the process of the development of the implementation program to enable researchers and practitioners to develop intervention programs tailored to their setting.

**Methods:**

We applied the intervention mapping (IM) method to develop a theory- and evidence-based intervention program to change vaccination behaviour among HCWs in nursing homes.

**Results:**

After a comprehensive needs assessment, we were able to specify proximal program objectives and selected methods and strategies for inducing behavioural change. By consensus, we decided on planning of three main program components, *i.e*., an outreach visit to all nursing homes, plenary information meetings, and the appointment of a program coordinator -- preferably a physician -- in each home. Finally, we planned program adoption, implementation, and evaluation.

**Conclusion:**

The IM methodology resulted in a systematic, comprehensive, and transparent procedure of program development. A potentially effective intervention program to change influenza vaccination behaviour among HCWs was developed, and its impact was assessed in a clustered randomised controlled trial.

## Introduction

Following 2004 guidelines by the World Health Organization, the Dutch association of nursing home physicians (Verenso) has been recommending influenza vaccination of healthcare workers (HCWs) [1]. In nursing homes, higher uptake of influenza vaccines has been associated with reduced morbidity and mortality among their frail patient population [2]. In a recent Cochrane review, an overall reduction in all-cause mortality of 32% (95% confidence interval 16 to 45%) was found in long-term care facilities in which part of the HCWs were vaccinated versus control homes. One of the studies from that review [3] revealed that in the control homes in a sample of 30 deaths 20% was caused by influenza. In the intervention homes none of the sampled deaths had evidence of influenza infection, which corresponds with a 100% reduction in deaths caused by influenza. In addition, Thomas *et al*. obtained an estimate of 29% reduction (95% confidence interval between 10 and 45%) in influenza-like illness in intervention homes as compared with control homes. It has been well established that during influenza epidemics, the etiological fraction of culture or PCR-confirmed influenza virus in elderly patients is high -- between 55% and 67% [4]. While immunisation of HCWs reduces the occurrence of influenza infections and associated productivity loss among the HCWs, it also ensures continuity of care during influenza epidemics [5-7]. A significant number of HCWs are infected with influenza each winter [8,9], and most of them continue to work despite of infection [5,10,11]. Therefore, HCWs can introduce influenza in healthcare settings and increase the risk of an influenza outbreak. Such an outbreak in turn can have significant consequences for patients and continuity of care in healthcare institutions such as those with long-term care, including nursing homes [9]. In The Netherlands, HCWs with risk-elevating conditions are routinely invited by their primary care physician. However, the majority of HCWs (approximately 85 to 90%) are otherwise healthy and despite recommendations to immunize this specific target group against influenza, vaccine uptake among HCWs in this high-risk setting remains far below the health objectives of 50% or more with estimated average vaccine uptake rates of 10% in 2005 [12].

To be most effective, implementation programs to change behaviour should be built upon a coherent theoretical base and should target all relevant determinants of influenza vaccine uptake among HCWs [13-15]. Previous programs targeting HCWs have, to our knowledge, not incorporated such a systematic approach. Often it remains unclear why specific interventions are chosen in implementation studies reported in the literature [16]. There are several more or less systematic methods available to develop implementation programs, including both exploratory methods (mainly based on brainstorming and consensus) and theory-based methods. One of these methods is the intervention mapping (IM) method, which offers a structured approach to develop theory- and evidence-based programs [17-19]. We used this IM method to systematically develop an intervention program to change vaccination behaviour among HCWs that could be implemented in nursing homes in the Netherlands. In an earlier report, we showed a mean 9% increase of vaccine uptake among HCWs in nursing homes that implemented the systematically developed program compared with control homes, with higher rates in those homes that implemented more program elements [20]. Here, we report in detail the process of the development of the implementation program to enable researchers and practitioners to develop intervention programs tailored to their setting.

## Methods

**Figure 1:**
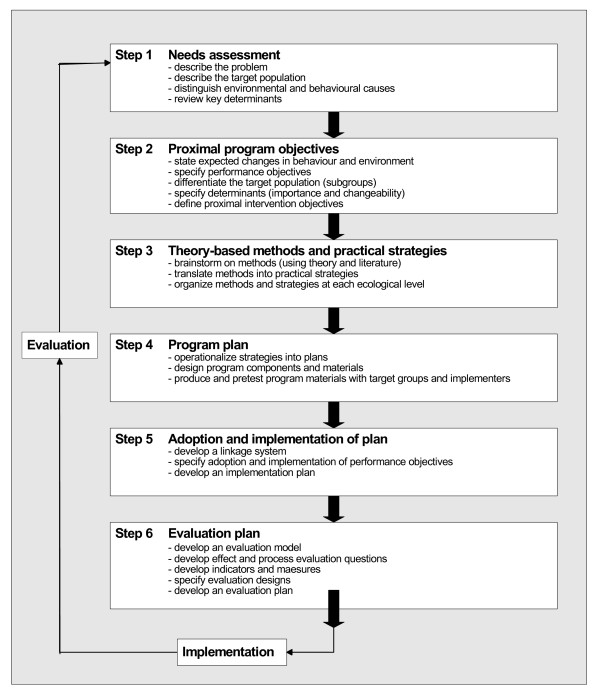
**Intervention mapping method (adapted from Bartholomew *et al*.)**[35].

The IM method is a framework developed in the field of health education and promotion to systematically design theory- and evidence-based health promotion programs [17]. It was originally developed for interventions aimed at high-risk behaviours (*e.g*., HIV prevention [18]), and has also been used for other types of interventions (*e.g*., quality improvement interventions [19]). The IM method follows several consecutive steps giving planners a systematic method for decision making in each phase of intervention development [17]. The process of intervention design can be divided into six steps: a needs assessment; specification of proximal program objectives; selection of theory-based methods and practical strategies for inducing change; planning the program; planning of program adoption and implementation; and planning for evaluation. The steps of the IM method and their components are shown in Figure 1.

### Developing the program

#### Step 1: needs assessment

To improve influenza vaccine uptake among healthcare workers (HCWs) of nursing homes, we first identified relevant barriers to and facilitators of vaccination uptake. These determinants may be related to the individual HCW or to the social, organisational, and economic context [16]. To explore all these levels, we organised three individual in-depth interviews with nursing home physicians and two focus group sessions (one with four nursing home physicians and one with three nursing assistants and two nurses). These were used to explore in a structured manner what determinants of influenza vaccination behaviour the participants experienced in daily practise. The structure of the sessions was based on both the theory of planned behaviour and the health behavioural model. Next, to complement these determinants identified by exploratory methods with theory-based determinants, we conducted an informal review of the international literature on determinants of influenza vaccine uptake among HCWs. Based on these qualitative methods, we conducted two quantitative questionnaire studies to specifically assess these determinants of vaccine uptake among HCWs in Dutch nursing homes.

### Questionnaire study one: Determinants of influenza vaccine uptake at management level

The first questionnaire study was conducted among the management of all 335 nursing homes in the Netherlands with an average size of 178 patients and 232 HCWs assessing organisational determinants at management level known from the literature to be associated with higher influenza vaccine uptake among their HCWs [12]. The response rate was 45%. In October 2005, the following items were assessed: uptake of influenza vaccination among patients and HCWs in the preceding season (2004 to 2005 season), whether the institution had a written policy on influenza vaccination for HCWs, what the current offering policy was (active request, employees initiative, or none), and if HCWs were currently offered information on influenza vaccination.

### Questionnaire study two: Determinants of influenza vaccine uptake at HCW level

The second study was a questionnaire study among HCWs of Dutch nursing homes assessing demographical, behavioural, and organisational determinants associated with uptake of influenza vaccination among HCWs. This questionnaire was based on the in-depth interviews and the focus group sessions, a review of the literature [21-28], and two previously developed questionnaires by our research group [29,30]. The questionnaire contained 12 questions on demographic determinants and 39 questions on behavioural determinants and on actual uptake of the vaccine. Questions on behavioural determinants were based on the 'Health Belief model' [31] and the 'Behavioural Intention Model' [32]. These models were selected because results of the in-depth interviews and focus group sessions indicated that most participants experienced determinants on this individual level. The following five Health Belief Model domains were assessed: perceived susceptibility, perceived severity, perceived benefits, perceived barriers, and cues to action. These were complemented with the two Behavioural Intention Model domains: attitude (including ethical views) and social influences. Finally, six questions assessed organisational determinants consisting of the current situation concerning organisation of information on influenza vaccination (information received or not, route of information, and whether information has been sufficient or not), opinion towards various routes of receiving information, and the current situation concerning the organisation of vaccine provision (if and how provision is organised and if this has been adequate). In December 2005, these determinants of influenza vaccination uptake were assessed by an anonymous, self-administered, 59-item questionnaire. In all, 1,125 of 1,889 questionnaires were returned from the 32 randomly selected study nursing homes. The mean age and gender distribution of study participants was similar to total HCW personnel in The Netherlands, but less educated staff appeared underrepresented, but we could take this determinant into account in the prediction model.

These two combined studies resulted in a total of 73 possible determinants of influenza vaccine uptake relevant for the development of the intervention program: 70 determinants on HCW level (12 demographical, 39 behavioural, and 19 organisational determinants) and three organisational determinants on management level.

#### Step two: Specification of proximal program objectives

To specify our intervention objectives, we analysed the relation between all 73 possible determinants (step 0) and actual vaccine uptake. The outcomes of our first study on management level with a response rate of 45% (149 out of 335 nursing homes) showed that having a written policy, actively requesting HCWs to get vaccinated, and informing HCWs about influenza vaccination were all associated with a significantly higher uptake of influenza vaccination among HCWs [12]. Mean differences (MD) of these three determinants were reported as measures of associations (see Table 1).

**Table 1:** Determinants resulting from the needs assessment and their importance and changeability

		Importance¹	Changeability^2^
**Determinants of influenza uptake at management level**			
a	Having a written policy	4.58	+
b	Actively requesting HCWs to get vaccinated	6.77	+
c	Informing HCWs about influenza vaccination	8.27	+

**Determinants of influenza uptake at HCW level**			
Demographical			
d	Presence of chronic illness	8.50	-
e	Working in health care for more than 15 years	2.32	-
Behavioural			
f	Perceived high personal risk	2.80	+
g	Perceived reduction of personal risk	2.56	+
h	Perceived reduction of risk to infect patients	3.29	+
i	Awareness of the existence of a guideline	1.86	+
j	Agreement with this existing guideline	2.75	+
k	Social influence of people close to the HCWs	5.33	-
l	Influence of media attention for avian influenza	2.24	+
m	All HCWs should get vaccinated	2.25	+
n	HCWs should get vaccinated because of their duty not to harm	4.71	+
Organisational			
o	Information received through an information meeting	3.40	+
p	Information received from a nursing home physician	2.11	+

^1 ^determinants at management level: importance defined by mean differences determinants at HCW level: importance defined by odds ratios

^2 ^-: not changeable, +: changeable

The outcomes of our second study on HCW level with data from 1,125 respondents (response rate 60%) enabled us to accurately predict influenza vaccine uptake on a HCW level based on a multivariate prediction model with 13 determinants (area under the receiver operating curve [AUC] of 0.95). This model included two demographical determinants, nine behavioural determinants, and two organisational determinants in which odds ratios (OR) were reported as measures of associations (see Table 1). The presence of chronic illness is a requirement for routine recommendations to be vaccinated by primary healthcare physicians in The Netherlands.

To quantify the 'importance' of the determinants resulting from both studies, we used the measures of association of the determinant with influenza vaccine uptake, *i.e*., the mean differences and odds ratios. We prioritised the importance of the determinants based on the strength of these associations (Table 1).

Next, to specify 'changeability,' we judged the changeability of the determinants based on consensus among all project group members (Table 1). Because the two demographical determinants (presence of chronic illness and working in healthcare for more than 15 years) were positively associated with influenza vaccine uptake, but not changeable, we did not define intervention objectives for these specific determinants nor used these determinants to define specific target subgroups.

Finally, the combination of importance and changeability of determinants was used to define intervention objectives (Table 2). For example, the determinant 'perceived high personal risk' was considered important (OR = 2.80) and changeable and therefore 'accomplishing awareness among HCWs of being at risk for an influenza infection and knowledge on the height of this risk' was defined as an intervention objective.

**Table 2:** Selected intervention objectives, methods and strategies

Determinants	Objectives	Methods and strategies
**Management level**		
Having a written policy	Stimulating nursing homes to develop a written policy on influenza vaccination of HCWs	Informing management on effect of a written policy (outreach visit, written information)
Actively requesting HCWs to get vaccinated	Actively requesting HCWs to get vaccinated	Executing the intervention program automatically leads to an active request
Informing HCWs about influenza vaccination	Having HCWs informed on influenza vaccination	Informing HCWs by plenary meetings, discussion in smaller groups, invitation letter, leaflets, posters, video, website

**HCW level**		
Presence of chronic illness	No objective set due to limited changeability	
Working in health care for more than 15 years	No objective set due to limited changeability	
Perceived high personal risk	Awareness among HCWs of being at risk for an influenza infection and knowing how high this risk is	- Provide risk information (plenary meeting, leaflets, website) - Evaluate own behaviour in smaller groups - Show a video with role-models
Perceived reduction of personal risk	HCWs being convinced that vaccination is effective in reducing the personal risk for an influenza infection	- Provide effectiveness information concerning reduction of personal risk (plenary meeting, leaflets, posters, website) - Interactive information provision by discussion in smaller groups - Show a video with role models
Perceived reduction of risk to infect patients	HCWs being convinced that vaccination is effective in reducing the risk to infect patients with influenza	- Providing effectiveness information concerning the reduction of infecting patients (leaflets, posters, website, plenary meeting) - Interactive information provision by discussion in smaller groups - Show a video with role-models
Awareness of the existence of a guideline	HCWs being aware of existence of guideline	Mention the existence of the guideline in program materials (leaflets, website, information meeting)
Agreement with this guideline	HCWs understanding reasoning of guideline	- Explain guideline (leaflets, website, plenary meetings) - Discuss the guideline in smaller groups
Social influence of people close to the HCWs	Also informing people close to the HCWs	Send a personal invitation letter for the plenary meetings to the home address of all HCWs together with an information leaflet
Influence of media attention for avian influenza	HCWs understand what avian influenza is and how it relates to annual human influenza	- Explain avian influenza on website
All HCWs should get vaccinated	HCWs understand the ethical aspects of influenza vaccination among HCWs	- Explain and discuss ethical aspects (leaflets, website) - Show a video with role-models - Discussion in smaller groups
HCWs should get vaccinated because of their duty not to harm	HCWs understand the ethical aspects of influenza vaccination among HCWs	- Explain and discuss ethical aspects (leaflets, website) - Show a video with role-models - Discussion in smaller groups
Information received through an information meeting	Conducting an information meeting	Execute an information meeting with plenary information on influenza and influenza vaccination and discussion in smaller groups
Information received from a nursing home physician	Having preferably a physician a local program coordinator	Nursing home physician signing invitation letters and shows his support during information meetings

#### Step three: Selection of methods and strategies

For the selection of theoretical methods and strategies we used the list of known types of implementation interventions from the EPOC data collection checklist [33]. In addition, we used both the general literature on the effectiveness of these different interventions [14] and the literature on previous studies that specifically tested intervention methods to increase influenza vaccine uptake among HCWs. A systematic review from 2006 evaluating whether promotional campaigns could improve uptake of influenza vaccination in HCWs was among the literature used [7]. Reviewing all available information, the project group finally decided on methods and strategies to be used in order to reach the intervention objectives (Table 2). For example, gender and presence of illness are associated with uptake, but cannot be changed. However, these determinants might be of use to define specific subgroups for the intervention. Perceived risk and potential reduction by vaccination can be changed by effective educational methods that focus on increasing knowledge like information leaflets, websites, group presentations, and videos with role models [13-15]. Ethical issues like 'do no harm' need to be targeted with more intensive activities such as small group discussions and role models in management of the centres. Social influence also asks for a more comprehensive approach that includes discussions at management level and discussion evoking items such as videos with role models [13-15]. Finally, logistics need to be worked out to reduce efforts to get the vaccine like introduction of mobile carts.

#### Step four: Planning of the program

Next, the methods described in Table 2 were operationalized into practical strategies and materials (Table 3). By consensus, the intervention program consisted of three main components. Component A included an outreach visit during which homes were to receive a step-by-step script of the program, all required materials, and background information on influenza vaccination of HCWs. The required materials consisted of announcements, a personal invitation letter, leaflets, posters, and the reference to the programs' website. Component B consisted of the plenary information meetings with a plenary presentation, discussion in smaller groups, and a video with role models. These meetings were to be organized by specialised nurses guided by a protocol. And, finally, component C prescribed the appointment of preferably a physician as a local program coordinator to organize and promote influenza vaccination.

**Table 3:** Components of the implementation program targeting determinants from Table 1

Component
A: Outreach visit during which the homes received:
• a step by step script of the program
• all required materials:
• announcement's (for the program, meetings and vaccination) [b,c,f,g,h,p]
• personal invitation letter for the meetings [b,c,k,p]
• information leaflets [b,c,f,g,h,i,j,k,m,n]
• posters [b,c,g,h]
• reference to the website: http://www.gepriktvooru.nl (in Dutch) [a,b,c,f,g,h,i,j,l,m,n,p]
• background information [a,i]

B: Two plenary information meetings with:
• plenary 1-hour presentation and discussion (see below) on influenza and influenza vaccination [b,c,f,g,h,o]
• discussion in small groups [b,c,f,g,h,i,j,h,k,l,m,n,o]
• a 10-minute video with role models [b,c,f,g,h,m,n,p]
• held by a specialised nurse of the local municipal health centre
• guided by a protocol

C: Appointment of preferably a physician as a local program coordinator:
• to organize and promote influenza vaccination [b,o]

[ ]: determinants integrated in program component indicated by corresponding letter from Table 1

All required materials were developed by our study group. The information leaflets and posters were developed in collaboration with the design department of the University Medical Center Utrecht and the information leaflet was pre-tested by three nursing assistants. They evaluated clearness, meaningfulness, and usefulness of the leaflet and were asked if any information was missing. The PowerPoint presentation for the information meetings, the leaflets, the posters, the video, and the website were all designed in a uniform style according to regulations of the University Medical Center Utrecht. For development of the website, we were assisted by our data management section. The video was recorded in a nursing home in Utrecht by a professional cameraman from the design department of the University Medical Center Utrecht. In the video, a nursing home physician, a nurse, and a patient shared their experiences on influenza and influenza vaccination with the viewers. The announcements and the personal invitation letter for the meetings were developed with standardised texts, leaving room to change dates, locations, and names according to the individual situation of nursing homes.

#### Step five: planning of program adoption and implementation

To assure program adoption, implementation, and sustainability, stakeholders were approached to give feedback on and to support the program. Representatives of the Dutch association of nursing home physicians (Verenso) and the association of nurses and nursing assistants (V&VN) were approached to judge the different elements of the program. The V&VN is the sole society for all Dutch nurses and nursing assistants with 36,000 members (approximately 10% of all HCWs in The Netherlands). They were asked for feedback on usefulness of the program elements and if program elements could be improved. This feedback was used to fine-tune the program elements, mainly by adjustment of difficulty of the language used. We did not translate the written information into other languages because most nurses in nursing homes understand the Dutch language. Support to the program was given byVerenso, V&VN, and two other relevant healthcare management associations (Sting and ActiZ) and visualised with their logo on program materials (*e.g*., the information leaflets). Furthermore, to support future implementation of the program -- without assistance by our study group -- a step-by-step script of the total program was developed. In addition, the plenary information meetings were held by specialised nurses of the local municipal health centre guided by a standardised protocol. In The Netherlands these municipal health centers have a supportive role in the prevention of infection prevention in general and specifically influenza. In this protocol, we also included a list of frequently asked questions and corresponding answers.

We planned to send all 335 Dutch nursing homes an invitation letter to participate in the program mid-2006. Nursing homes who responded positive to this invitation (n = 33) would then be asked to appoint preferably a physician as local program coordinator (component C). Next, all nursing homes would be visited in September to deliver the step-by-step script of the program, all required materials, and the background information on influenza vaccination of HCWs (component A). Following these visits, execution of all program activities would be planned for October and November prior to the actual immunization of HCWs. During this period, the plenary information meetings were to be held by a specialised nurse of the local municipal health centre (component B).

#### Step six: Planning for evaluation

Our evaluation plan included both an effect and a process evaluation. We planned to evaluate the effectiveness of the program on influenza vaccine uptake among HCWs in Dutch nursing homes by comparing uptake in a group of at least 12 nursing homes randomly allocated to receive the intervention program with the uptake in a similar number of control homes. For this, we planned to perform a clustered randomised controlled trial [33]. In the trial, 33 nursing homes participated with a total of 6,636 HCWs. Mean number of patients per home were 160 and 200 HCWs. Percentage of females was 90% and mean age was 40 years. The mean vaccine uptake in both intervention and control homes was 11% at baseline in 2005. In all, these figures were similar to The Netherlands as a whole. Furthermore, we decided to measure compliance with the programme components (process evaluation). For this purpose, we planned to register whether nursing homes were visited, whether plenary information meetings were organised, and how many HCWs visited these meetings, what the profession of the local program coordinator was, and, finally, all costs related to the program. In this manner we could, on the one hand, explore whether compliance with the components of the program influenced the effectiveness of the program. This information can be used to, if necessary, adapt the program. On the other hand, we could use this information to estimate program costs.

## Discussion

This paper presents the process by which a theory- and evidence-based intervention was developed to improve influenza vaccination behaviour among HCWs. The IM method was used to systematically develop this intervention.

A major strength of the developing process was the comprehensive and thorough needs assessment that clearly identified relevant determinants for influenza vaccine uptake among HCWs on both management and HCW level. Combining explorative and theory-based methods assured that determinants not anticipated beforehand were included in the needs assessment and helped broaden the scope of this needs assessment. Based on the results of the needs assessment, we were able to quantify the importance of the determinants. This provided an anchor for the specification of program objectives and the successful further development of the intervention.

The selection of methods and strategies (step three of the IM method) is challenging, because specific objectives can ask for a variety of different interventions, and consensus is needed by the developers on the intervention with presumed highest impact [14]. As yet, there is no firm guidance on what interventions should be linked to what specific objectives. To facilitate this process, we considered the 'Health Belief model' [31] and the 'Behavioural Intention Model' [31], and the available evidence regarding the effectiveness of interventions [14,33] combined with common sense and creativity to reach consensus.

Our systematic, comprehensive, and transparent description of all the steps in the development of the program enables future users to assess and adapt the program where necessary or to replicate the steps described when developing a similar program for a different population. Applying the systematic, comprehensive, and transparent IM approach guided and facilitated our development process. It may seem elaborate and time consuming, but a review of health promotion intervention studies has shown that the quality of planning is important for the success of the intervention [34,35]. Recently, we published our paper on the effects of the randomized controlled trial of this developed intervention program [20]. We showed that the intervention program resulted in a significantly higher (25% in intervention group versus 16% in control group), though moderate, influenza vaccine uptake among HCWs in nursing homes.

## Competing interests

The authors declare that they have no competing interests.

## Authors' contributions

ILvdA conducted the questionnaire studies and wrote the paper. MEH supervised the design of the program and drafts of the paper. TJMV commented on the paper. JRD critically commented on the paper and revised earlier drafts. JJvD and EH contributed both equally to the design of the program, were involved in earlier drafts, supervised the project and final draft. All authors have read and approved the final manuscript.
